# The Correlation of Microstructure and Mechanical Properties of In-Situ Al-Mg_2_Si Cast Composite Processed by Equal Channel Angular Pressing

**DOI:** 10.3390/ma12091553

**Published:** 2019-05-12

**Authors:** Mahdi Chegini, Mohammad Hossein Shaeri, Reza Taghiabadi, Sajjad Chegini, Faramarz Djavanroodi

**Affiliations:** 1 Department of Materials Science and Engineering, Imam Khomeini International University (IKIU), Qazvin 3414916818, Iran; mchegini88@gmail.com (M.C.); Taghiabadi@eng.ikiu.ac.ir (R.T.); 2 Department of Materials Science and Engineering, Shahid Bahonar University (SBU), Kerman 7616913439, Iran; sajjadcheginy@gmail.com; 3 Mechanical Engineering Department, Prince Mohammad Bin Fahd University, Al Khobar 31952, Saudi Arabia; f.djavanroodi@imperial.ac.uk; 4 Department of Mechanical Engineering, Imperial Collage London, London SW7, UK

**Keywords:** Al-Mg_2_Si composites, ECAP process, microstructure, mechanical properties, density, porosity

## Abstract

In this paper, the effect of equal channel angular pressing (ECAP) on microstructure and mechanical properties of hypereutectic Al-20%Mg_2_Si and Al-15%Mg_2_Si, as well as hypoeutectic Al-10%Mg_2_Si composites has been investigated. After fabricating the composites by in-situ casting, the composites were processed using the ECAP process up to two passes at room temperature. Microstructural studies have been carried out using a field emission scanning electron microscopy equipped with an energy dispersive X-ray spectrometer. Mechanical properties were also documented using Vickers microhardness and shear punch tests. In the hypereutectic composites, a decrease in the average size of pro-eutectic Mg_2_Si (Mg_2_Si_p_) particles, breakages in eutectic networks, and lengthening of the Al (α) phase in direction of shear bands were observed after the ECAP process. For instance, the average size of Mg_2_Si_p_ Particles in Al-20%Mg_2_Si composite reduced from 40 to 17 μm after 2 passes of ECAP. Furthermore, a uniform distribution of Mg_2_Si_p_ particles was developed in the matrix. In hypoeutectic composite, the ECAP process caused a uniform distribution of eutectic Mg_2_Si (Mg_2_Si_E_) in the matrix that considered a favorable microstructure. Microhardness measurements and shear punch results showed an ascending trend after each pass of ECAP for all specimens. For example, microhardness and shear strength of Al-20%Mg_2_Si increased from 88 HV and 109 MPa to 119 HV and 249 MPa after two passes indicating 35% and 34% increments, respectively. Density and porosity calculations by Archimedes principle revealed that the density of the composites increased after two passes of ECAP due to the reduction of porosity.

## 1. Introduction

In situ particulate Al-Mg_2_Si composites have attracted more attention because of their improved properties, such as low density, good wear resistance, and excellent castability [1]. These composites are considered as new engineering materials and may be reliable alternatives for some alloys in automobile and aerospace industries. In situ metal matrix composites have some advantages like uniform distribution of the reinforcing phase, good particle wetting, and low production costs. These composites have high potential as a wear resistant material due to the presence of Mg_2_Si intermetallic compound which has a high melting temperature (1085 °C), low density (1.99 × 10^3^ Kg m^−3^), high hardness (4500 MN m^−2^), and low thermal expansion coefficient (7.5 × 10^−6^ K^−1^) [2]. 

Unfortunately, Mg_2_Si particles tend to coarsen in the cast composites during solidification. Therefore, several researchers have been focused on the modification of microstructure especially the size and morphology of Mg_2_Si particles. There are some methods that can be implied for microstructural modification, such as chemical modification, thermomechanical process, and heat treatment [3]. The addition of Si [3], Cu [4], Ni [5], Sr [6], Li [7], TiB_2_ [8], Cr [9], La [10], Na [11], Ce [12], and Y [13] elements to Al-Mg_2_Si composites has been investigated previously. Furthermore, the influence of solution heat treatment at various temperatures has been also studied [14]. The effects thermo-mechanical processes such as hot extrusion on hypereutectic and hypoeutectic composites have been investigated by Emamy et al. [15]. In addition, the influence of lithium and cooling rate [16], phosphorus and type of mold [17], as well as titanium and hot extrusion [18] on microstructure and mechanical properties have been studied in previous works. Some other techniques have been employed to achieve a better microstructure, and as a consequence, higher mechanical properties, such as the semi-solid process [19], superheating melt treatment [20], and ultrasonic stirring [21,22].

Severe plastic deformation processes (SPD) are referred to as methods of forming, in which high strains introduced to the specimen for production of ultrafine grains (UFG) in such a way that no significant changes occur in the overall dimensions of the specimen [23,24]. One of these methods is equal channel angular pressing (ECAP) which was first introduced by Segal in the 1980s. Materials with grain sizes of a few hundred nanometers can be produced through the ECAP process. The intensified shear stresses imposed on the material during the ECAP process increases the density of dislocations. Subsequently, these dislocations form low-angle grain boundaries (LAGB), which ultimately produce rows of fine grains by converting to high-angle grain boundaries (HAGB) [24,25,26,27,28,29,30].

According to previous studies, a few studies have been conducted on the effects of SPD processes on hypoeutectic Al-Mg_2_Si composites, and no research has been conducted on hypereutectic Al-Mg_2_Si composites. Considering that the SPD methods, including ECAP, can have significant effects on the microstructure improvement, and as a result, the increment of the mechanical properties of this highly applied engineering composites, investigating the ECAP process of Al-Mg_2_Si composites can be worthy. The aim of the current study was to investigate the effect of the ECAP process on modifying the microstructure and also improving the mechanical properties (hardness and shear strength increment) of Al-Mg_2_Si composites (Hypoeutectic and hypereutectic).

## 2. Materials and Methods 

In order to produce in-situ Al-Mg_2_Si composites, pure commercial Aluminum, Silicon and Magnesium were used. The values of the elements were calculated and weighed according to the optimum final composite composition (20, 15 and 10% Mg_2_Si). In order to prepare the desired compound, Aluminum was first placed in a clay-graphite crucible in a resistance furnace. After full melting of Aluminum and superheating to 780 °C, Silicon and Magnesium were added to the melt, respectively. When the dissolution took place, the melt was poured into the steel mold after slag-removal. The chemical composition of the composites was measured by the GNR Italy Metallab-7580j (G.N.R. S.r.l, Agrate Conturbia, Novara, Italy) spectrometer (Table 1). After casting, the rods with a length of 100 mm and a diameter of 12 mm were prepared by machining for ECAP process.

In order to carry out the ECAP process, a die with two identical channels with intersecting angle (Φ) of 90° was used. The external curvature angle at the contact of the two channels (Ψ) was 20°. The strain produced by this die was equal to 1 at each pass of ECAP. A hydraulic press with a piston speed of 1.5 mm/s and a capacity of 100 tones was employed to apply ECAP force. Molykote 1000 paste lubricant (Dow Corning, Midland, MI, USA) was used to reduce friction between the specimen and die. Furthermore, in order to reduce adhesion and friction during ECAP process, specimens were inserted in copper tubes. In addition, to prevent cracking of the specimens, a back pressure was imposed to the specimens by using a rod in front of them. ECAP process was performed at room temperature with route A (no rotation between each pass).

Cast Al-Mg_2_Si composites with different amounts of Mg_2_Si were successfully pressed up to two ECAP passes, but after the third pass cracks were observed in the specimens and during the fourth pass, the specimens were broken. Therefore, microstructural and mechanical properties studies were carried out on the initial specimen as well as one pass and two passes ECAPed specimens. In order to study the microstructure of the specimens, the specimens were sectioned from the middle. Then, the specimens’ cross sections were mounted and polished with standard methods of preparation. For evaluating the microstructure of the hypereutectic and hypoeutectic composites, hydrofluoric (2 mL of hydrofluoric acid and 98 mL of distilled water) and Keller etchants (2 mL of hydrofluoric acid, 3 mL of chloride acid, 5 mL of nitric acid and 190 mL of distilled water) were used, respectively. In order to investigate the microstructure, chemical analysis of the phases and three-dimensional observation of precipitated phases a field emission scanning electron microscopy (FESEM, TESCAN BRNO, Kohoutovice, Czech Republic) was employed. Chemical analysis of the precipitated phases was studied using energy dispersive spectroscopy (EDS, TESCAN BRNO, Kohoutovice, Czech Republic). Microstructural parameters such as the size of particles, the volume fraction of phases and grain size were calculated using Image J software. It should be noted that for calculating the microstructural parameters, at least three images were analyzed and the average magnitudes were reported alongside with their standard deviation. 

Density and porosity measurements were performed using a digital density meter with an accuracy of 10^−2^. Archimedes principle was used to calculate the density and porosity percentage. For this purpose, the initial weights of specimens were measured (*M_d_*). Then the specimens immersed in distilled water (*M_w_*) were weighed. Subsequently, density and porosity were computed by the following equations:

(1) $$\rho_{exp}=\frac{M_{d}}{M_{d}-M_{w}}$$

(2) $$Porosity\;\left(\%\right)=\frac{100\left(\rho_{exp}-\rho_{t}\right)}{\rho_{exp}}$$

where $\rho_{exp}$ and $\rho_{t}$ are experimental and theoretical densities, respectively.

The hardness of the specimens was measured by Vickers microhardness machine (Koopa MH1, Koopa Co., Sari, Mazandaran, Iran) at 1000 g force and 10 s dwell time, according to ASTM-E384 standard [Standard Test Method for Microindentation Hardness of Materials, ASTM International, West Conshohocken, PA, USA, 2017]. The test was repeated at least 10 times for each specimen and the averages values were reported along with their standard deviation.

Shear punch tests were performed using Zwick/Roel Z100 universal testing machine (ZwickRoell GmbH & Co., Ulm, Germany). For shear punch tests, no lubricant was used. The amount of applied force in terms of punch displacement was measured and the stress in MPa was calculated using the following equation [31,32,33]:

(3) $$\tau =\frac{P}{\pi dt}$$

where *P* is the shear punch force in Newtons, *t* is the sample thickness in millimeters, and *d* is the mean diameter of the punch and mold in millimeters. The shear punch test graphs were obtained by plotting shear stress in terms of normal displacement. Normal displacement was obtained from the following equation [31,32,33]:

(4) $$d=\frac{h}{t}$$

where *d* is the normal displacement and *h* is the punch displacement in millimeters. The test was repeated three times for each specimen, and difference in shear yield strength and ultimate shear strength was less than 5 percent. The tests were also performed at a speed of 0.001 mm/s.

## 3. Results and Discussion

### 3.1. Microstructure

**Figure 1:**
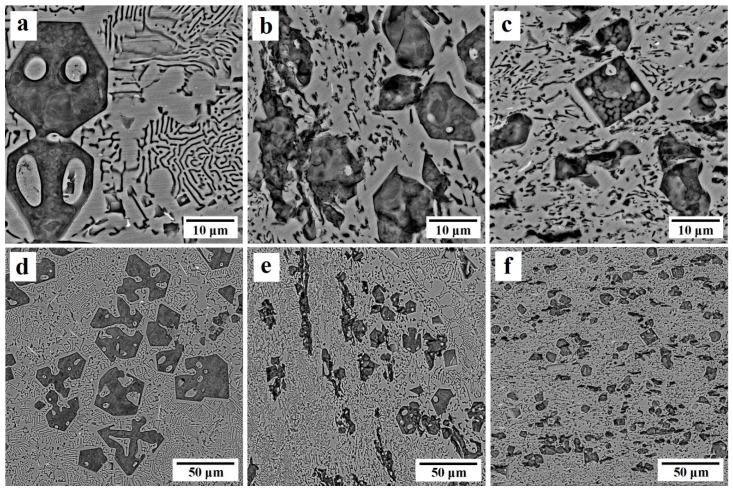
FESEM images of Al-20%Mg_2_Si composite; (**a**,**d**) before the equal channel angular pressing (ECAP) process, (**b**,**e**) after the first pass, and (**c**,**f**) after the second pass of ECAP.

FESEM images of the Al-20%Mg_2_Si composite, before and after ECAP, are shown in Figure 1. According to Figure 1a,d, the initial microstructure consists of binary eutectic phase and pro-eutectic Mg_2_Si (Mg_2_Si_p_) particles (black phase) that possessed irregular, dendritic and rough morphology with sharp corners. The distribution of this phase is not homogenous and uniform, and the particle size varies from 30 to 129 μm. The Al (α) phase (gray phase), which is an aluminum solid solution containing silicon and magnesium, was formed around Mg_2_Si_p_ particles or independently in the matrix. The binary eutectic has an alternative layered structure of Al (α) and eutectic Mg_2_Si (Mg_2_Si_E_). The interface between Mg_2_Si_p_ and matrix is faceted. As can be seen, some holes are presented in most of the Mg_2_Si_p_ particles. The formation of hopper-like crystals is attributed to undisturbed melt during growth so that the aluminum-rich blanket over the facets becomes thicker with time. Thus, layers nucleated at the corners suffering from sufficient Mg_2_Si supplies. Consequently, the corner growth continues, while at the face center, the growth in [111] direction is limited by the thickening of the aluminum-rich blanket. This phenomenon usually occurs at the later stages of solidification, in which the melt becomes more viscous and melt movement is limited [17]. 

**Figure 2:**
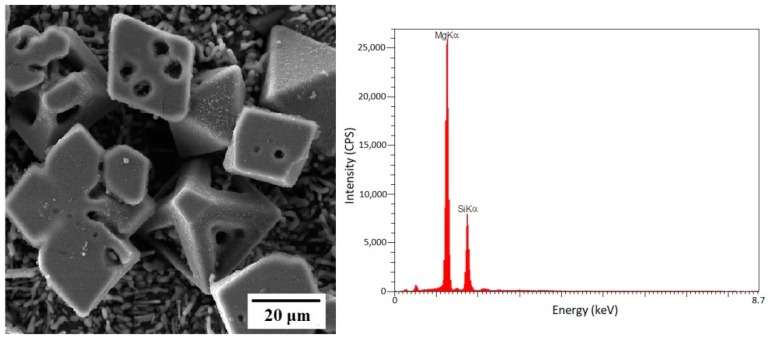
Three-dimensional FESEM image and EDS analysis of Mg_2_Si_P_ phases in Al-20%Mg_2_Si composite.

EDS results and three-dimensional images of observed particles in Al-20%Mg_2_Si composite are presented in Figure 2. In this composite, intermetallic Mg_2_Si precipitated in two different modes. The first one is Mg_2_Si_p_ particles, which exists as an initial phase in hyper-eutectic Al-Mg_2_Si composites. The second one is the Mg_2_Si_E_ layers, which is present in all Al-Mg_2_Si composites studied in current research as a two-phase eutectic with two types of morphology (layer and rod-like morphology).

After the first ECAP pass, some dendritic Mg_2_Si particles and a portion of the binary eutectic were broken and elongated along the shear bands. The Al(α) phase was also elongated in the direction of shear bands (Figure 1e). After the second pass, the intensity of the shear stress in shear bands was enhanced and its effect on the microstructure modification increased. Consequently, further Mg_2_Si_P_ was broken and their redoubled movement in the direction of the shear bands caused a more uniform distribution of particles in the matrix (Figure 1f). Most parts of the binary eutectic phase were also broken and the particles were distributed uniformly in the Al(α) phase. 

**Figure 3:**
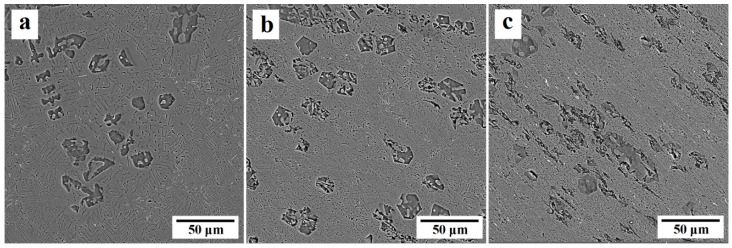
FESEM images of Al-15%Mg_2_Si composites; (**a**) before the ECAP, (**b**) after the first pass, and (**c**) after the second pass of ECAP.

Figure 3 depicts the microstructure of Al-15%Mg_2_Si composites before and after ECAP process. As can be seen, similar to the Al-20%Mg_2_Si composite, the microstructure contains Mg_2_Si_P_ with a rough and dendritic morphology with hopper-like crystals in large particles. Al-20%Mg_2_Si and Al-15%Mg_2_Si are both hypereutectic composites; therefore, both of them contain Mg_2_Si_p_. The amount of Mg_2_Si_p_ in Al-15%Mg_2_Si composite is lower than that of Al-20%Mg_2_Si composite as a result of the lower amount of Mg_2_Si in the composition of the composite. By reducing the volume fraction of Mg_2_Si_P_ particles, their average size was also decreased. The Mg_2_Si_P_ particles distributed heterogeneously in the matrix with a size ranging from 20 to 100 μm. After the first ECAP pass, the Mg_2_Si_p_ particles which were located on the shear bands path were broken and oriented along the shear direction. Meanwhile, the Al(α) phases were elongated in the direction of shear bands, and some portions of the binary eutectic phase located in the direction of shear bands were broken. After the second ECAP pass, the refinement of the microstructure was developed in the composite, and due to movement of the broken arms of dendrites in the direction of the shear bands, a more uniform distribution of Mg_2_Si_P_ particles in the matrix of the composite was developed. 

**Figure 4:**
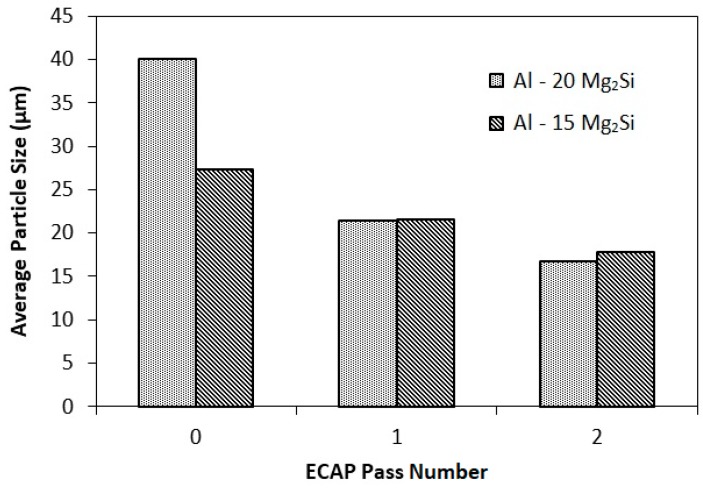
Variations of the Mg_2_Si_P_ particles’ average size in Al-20%Mg_2_Si and Al-15%Mg_2_Si composites as a function of ECAP pass number.

Figure 4 shows the variations in the average size of the Mg_2_Si_P_ particles. The average sizes of the Mg_2_Si_P_ particles in the cast Al-20%Mg_2_Si and Al-15%Mg_2_Si composites were about 40 and 27 μm, respectively. The average sizes of the Mg_2_Si_P_ particles in the Al-20%Mg_2_Si composite has already been measured by Emamy et al. [15] in the range of 70–80 μm. In another study, Hadian et al. [16] studied the effect of sample diameter on the average size of Mg_2_Si_P_ particles. They have found that by decreasing the sample size, the cooling rate increases. Accordingly, Mg_2_Si_P_ particles would not have enough time to grow and consequently the average size decreases. Influence of casting mold type has been studied by Qin et al. [17]. Results show that using permanent casting molds such as steel reduces the average size of Mg_2_Si_P_ particles due to the higher chilling capacity. Therefore, it can be concluded that difference in the calculated average size of the Mg_2_Si_P_ particles in current research with previous works lies in the differences in the diameter of the casting sample and type of the molds. After the first ECAP pass, the average size of Mg_2_Si_P_ particles in the Al-15%Mg_2_Si composite was reduced by 47% to 21 μm, and after the second pass, it was reduced by 22% to about 17 μm. In the Al-20%Mg_2_Si composite, after the first and second ECAP passes, the average size of Mg_2_Si_P_ particles was decreased by 21 and 17% to about 22 and 18 μm, respectively.

**Figure 5:**
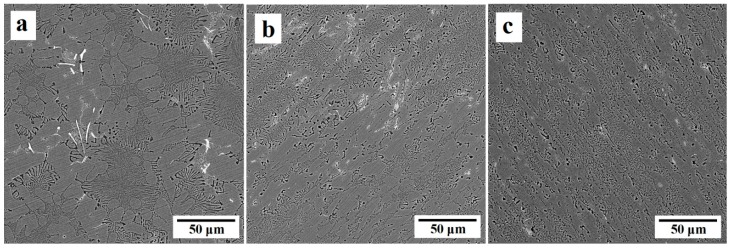
FESEM images of Al-10%Mg_2_Si composite; (**a**) before the ECAP process, (**b**) after the first pass and (**c**) after the second pass of the ECAP process.

FESEM images of initial and ECAP-processed Al-10%Mg_2_Si composite are presented in Figure 5. It is obvious that Mg_2_Si_P_ particles do not exist in the matrix, and the microstructure consists of the Al(α) as the primary phase with round morphology (non-faceted interface) and binary eutectic (Al(α) + Mg_2_Si_E_) with layered morphology (faceted, non-faceted interface). Some impurity of iron (white phase) is also present in the microstructure which is from the raw materials or the tool used in the casting process. A noteworthy point about the eutectic phase is the difference between the spaces of eutectic layers in different parts of the composite, as a result of the various freezing temperature [14]. After the first ECAP pass, the Al(α) phase was elongated in the direction of the shear bands, and length to width ratio was increased. Some portions of the binary eutectic phase located in the path of the shear bands were also broken and oriented in their direction. After the second pass, due to the greater intensity of the shear stresses within shear bands, the ratio of length to width of Al(α) phase was increased. In addition, more portions of the binary eutectic phase were broken, so Mg_2_Si_E_ phase was more uniformly distributed in the matrix.

### 3.2. Density and Porosity Percentage

**Figure 6:**
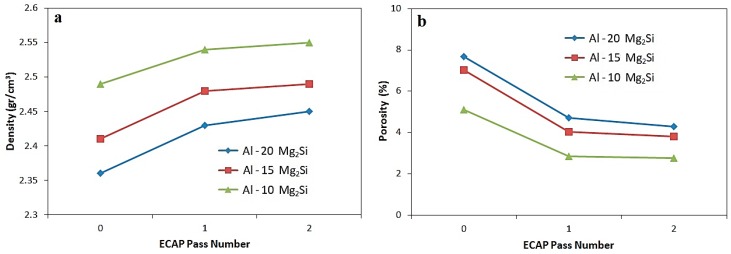
The variation of density and porosity percentage of Al-Mg_2_Si containing 10, 15 and 20% Mg_2_Si as a function of ECAP pass number; (**a**) density and (**b**) porosity percentage.

Figure 6 displays the variation of density and porosity percentage of casting Al-Mg_2_Si composites with different amount of Mg_2_Si, as a function of ECAP pass number. The theoretical densities of composites containing 10, 15, and 20% Mg_2_Si calculated by rule of mixtures were 2.62, 2.558, and 2.55 gr/cm^3^, respectively. As can be seen, after the first ECAP pass, the density of all three composites was increased significantly, which is due to the decrease in porosity amount and reduction in the size of the voids in the composites. On the other hand, there was no significant change in the density of the composites after subsequent passes [15]. Furthermore, increasing the Mg_2_Si amount in the composites led to an increment in porosity amount owing to the presence of more Mg_2_Si_P_ particles in the matrix. 

### 3.3. Mechanical Properties

**Figure 7:**
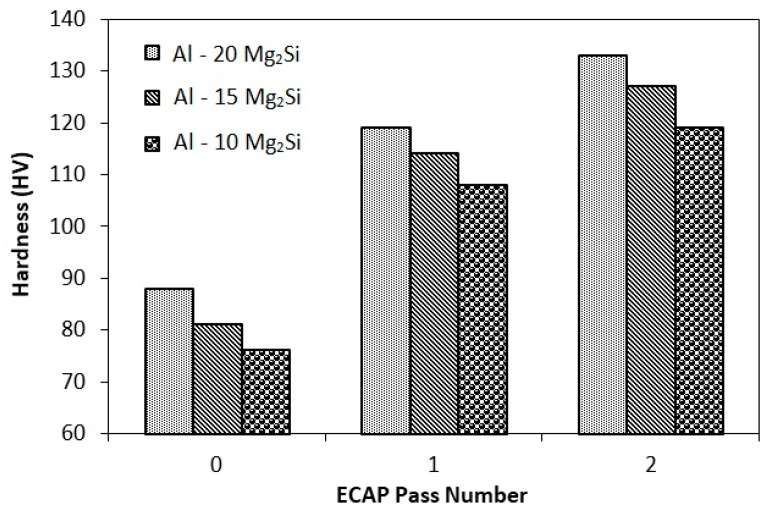
The Vickers’ microhardness (HV) of the Al-Mg_2_Si composites with different amount of Mg_2_Si, as a function of the number of ECAP passes.

Figure 7 illustrates the Vickers’ microhardness (HV) of the Al-Mg_2_Si composites with different amount of Mg_2_Si, as a function of ECAP pass number. As shown, increasing ECAP passes causes an enhancement in hardness value of all composites. After the final pass of ECAP, the hardness values of composites containing 10, 15, and 20% Mg_2_Si were about 61, 57, and 51 HV, respectively. According to hardness diagrams, this increment in the first pass is more profound in comparison with that of the subsequent passes. The hardness increment after the second pass in all three composites was less than 10%. The improvement of microhardness during ECAP is attributed to various mechanisms such as reduction of porosity and microstructural defects, the interaction between dislocations (work hardening), grain refinement of the matrix and modification of Mg_2_Si_P_ particles and binary eutectic morphology. The high strain imposed to the composites in the first pass of ECAP cause considerable work hardening, and accordingly, the hardness increases notably in the first pass, while by increasing the number of ECAP passes, the work hardening capability of the composites reduces, and consequently the hardness increases slightly. 

It is also clear from Figure 7 that the hardness of the composites increases with increasing Mg_2_Si content, which is very hard reinforcement. On the other hand, the highest hardness increment after ECAP process occurred in Al-10%Mg_2_Si composites (42%). This can be attributed to the possibility of micro-cracks formation during ECAP process of composites containing Mg_2_Si_P_ particles, as well as greater effect of ECAP process on modification of eutectic and primary Al(α) phase in the Al-10%Mg_2_Si composite.

**Figure 8:**
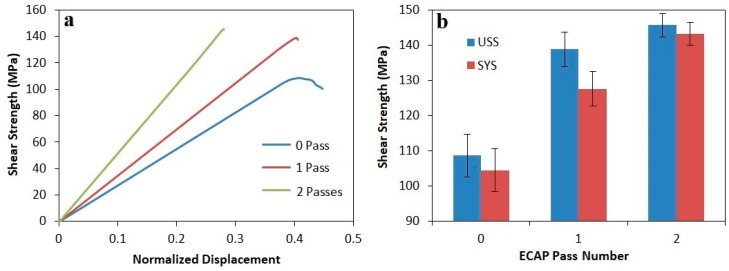
(**a**) The shear stress-normalized displacement curve of Al-20%Mg_2_Si composite before and after the ECAP process. (**b**) Ultimate shear strength and shear yield strength diagrams of Al-20%Mg_2_Si composite as a function of the number of ECAP passes.

**Figure 9:**
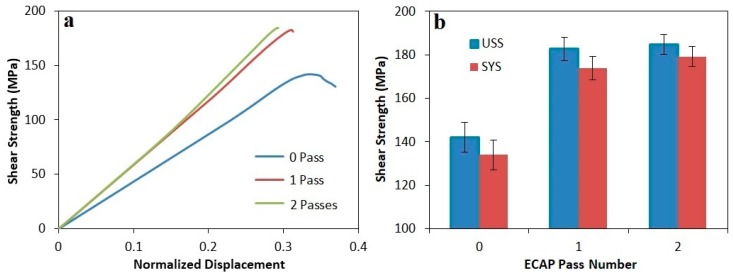
(**a**) The shear stress-normalized displacement curve of Al-15%Mg_2_Si composite before and after the ECAP process. (**b**) Ultimate shear strength and shear yield strength diagrams of Al-15%Mg_2_Si composite as a function of the number of ECAP passes.

**Figure 10:**
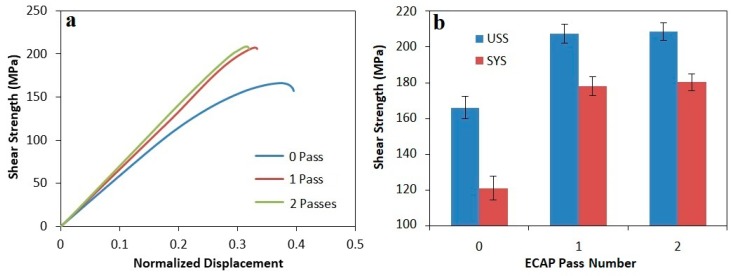
(**a**) The shear stress-normalized displacement curve of Al-10%Mg_2_Si composite before and after the ECAP process. (**b**) Ultimate shear strength and shear yield strength diagrams of Al-10%Mg_2_Si composite as a function of the number of ECAP passes.

The shear stress-normalized displacement curves and the corresponding ultimate shear strength (USS) and shear yield strength (SYS) of all three composites before and after ECAP process are presented in Figure 8, Figure 9 and Figure 10. As can be seen, after the final ECAP pass, the ultimate shear strengths of composites containing 20, 15, and 10% Mg_2_Si were increased by about 34, 29 and 25%, respectively; the shear yield strength was also enhanced by about 38, 34 and 49%, respectively. According to diagrams, the most considerable part of the strength increment occurred after the first pass, and the strength increment after the second pass of ECAP was negligible. This issue has also been observed in previous investigations after ECAP process of various materials [28,34,35]. In all three composites, the increment of the shear yield strength after ECAP process was more than the ultimate shear strength, which indicates a reduction in work hardening rate after ECAP process. Due to high degree of the material’s work hardening during ECAP process, their work hardenability would be reduced afterward [26]. Another noteworthy point is further increment in strength, softness (normal displacement) and work hardening rate of hypoeutectic composite (containing 10% Mg_2_Si) compared to hypereutectic composites (containing 15 and 20% Mg_2_Si). This is due to the presence of Mg_2_Si_P_ particles, and also increasing their volume fraction. Therefore, because of the brittle nature of these particles, the probability of cracking and breaking during the shear punch test is greater and affects the mentioned properties.

Principally, increasing the strength of Al-Mg_2_Si composites during ECAP process is related to the effect of three different strengthening mechanisms. The first mechanism is work hardening caused by increment of dislocations as well as considerable grain refining during ECAP process, which improve the strength of the composites in accordance with Taylor and Hall-Petch equations, respectively [35,36,37,38]. The second mechanism is the reduction of Mg_2_Si_P_ particle size and modification of Mg_2_Si_P_ and eutectic phase during ECAP process. As already mentioned, during ECAP process, Mg_2_Si_P_ particles were broken up and their morphology was modified and distributed more uniformly. Likewise, the morphology of the Mg_2_Si_E_ Phase was changed from a layer-like structure into a point-like and flake-like structure and distributed uniformly in the matrix. Morphological modification of the intermetallic phases makes them more difficult barriers against dislocations movement, and thus improves the strength. In addition, in brittle fracture of metal matrix composites, cracks, and local stress concentrations along the interface of particles and matrix cause failure at extremely lower stress than their intrinsic yield strength. The Mg_2_Si_P_ particles with local stress concentrations such as sharp corners and internal holes which have been mentioned in microstructure section can act as crack initiation point and encourage their growth and propagation. Therefore, it is expected that morphological modification of the Mg_2_Si_P_ particles and producing a finer microstructure leads to more fracture energy toleration and higher strength in composites [9]. The third mechanism is a reduction of porosity and structural defects, and consequently of the increment of composites’ density during the ECAP process. Porosities and structural defects are vulnerable sites for crack initiation. Consequently, the reduction of these defects during the ECAP process can improve the composites’ strengths.

In previous research by Bian et al. [39], the effect of the ECAP process on Al-10%Mg_2_Si composites has investigated. In this study, the composite’s ultimate tensile strength (UTS) has increased to about 210–217 MPa after four passes of ECAP in various routes. In another research by Emamy et al. [15], after hot extrusion process at 500 °C, the UTS of Al-15%Mg_2_Si and Al-20%Mg_2_Si composites has reached to 225 and 221 MPa, respectively. Considering that in accordance with Tresca criterion, the tensile to shear strength ratio of metals is 2 [31,32], and the previous research by Chegini et al. [40] has found that in Al-Mg_2_Si composites containing different amounts of Mg_2_Si, the tensile to shear strength ratio is higher than 2. Therefore, it can be concluded that the strength obtained after 2 passes of the ECAP process in the current study is significantly better than the values obtained in previous studies. This shows a great ability of the ECAP process for improving the mechanical properties of Al-Mg_2_Si composites.

## 4. Conclusions

In the present study, the effects of severe plastic deformation by the ECAP process on microstructure and mechanical properties of hypereutectic composites (Al-20%Mg_2_Si and Al-15% Mg_2_Si) and hypoeutectic composite (Al-10%Mg_2_Si) have been studied. Microstructure was characterized by FESEM, and mechanical properties were studied using Vicker’s microhardness and shear punch tests. The density and porosity of composites were also calculated in accordance with the Archimedes principle. The main conclusions of current research are as follows:

(1) The results obtained from FESEM study showed that in the hypereutectic composites, after each pass of ECAP process, Mg_2_Si_P_ particles and binary eutectic phase were broken and elongated along the shear bands direction. Consequently, a uniform distribution of Mg_2_Si_P_ particles was created in the matrix. For example, in Al-20%Mg_2_Si composite, the average size of the particles decreased from 40 to 17 μm. On the other hand, the binary eutectic networks were broken up and the Al(α) phase was oriented along shear bands in both hypereutectic and hypoeutectic composites, and accordingly, a uniform distribution of Mg_2_Si_E_ was made in the matrix.

(2) The hardness measurement results showed an increment in all composites after each pass of ECAP. The hardness increment after the first pass was more than subsequent passes. For example, the hardness of the Al-10%Mg_2_Si composite was increased by about 42% (from 76 HV to 108 HV), and 10% (from 108 HV to 119 HV) after the first and second pass of ECAP, respectively.

(3) The shear punch test results indicated that shear yield strength and ultimate shear strength of all composites enhanced after each pass of ECAP. After the first pass, the highest increment was recorded, and in subsequent passes, the increasing trend was continued with a slight slope. The difference between USS and SYS after the first pass was decreased sharply, indicating a reduction in formability after the ECAP process. 

(4) The results of density and porosity calculations in accordance with Archimedes principle showed high porosity content and low density of the cast composites compared to that of the theoretical density. After the ECAP process, the porosity of the composites decreased, and consequently, the density increased. The highest increment in density and reduction of porosity was after the first pass, which could be due to the decrease in the diameter of porosities and voids to a critical radius under pressure, while their sizes cannot be lowered by continuing the pressing process during subsequent passes.

## Figures and Tables

**Table 1:** Chemical composition of Al-Mg_2_Si composites in the present paper (wt.%).

Material	Mg	Si	Fe	Ni	Zn	Ca	Cu	Ti	Al
Al-10% Mg_2_Si	6.63	4.04	0.16	0.004	0.06	0.02	0.001	0.003	Bal.
Al-15% Mg_2_Si	9.82	5.72	0.17	0.003	0.01	0.03	0.002	0.004	Bal.
Al-15% Mg_2_Si	12.8	7.7	0.19	0.005	0.01	0.04	0.003	0.006	Bal.
